# Gravity-dependent change in the ‘light-from-above’ prior

**DOI:** 10.1038/s41598-018-33625-2

**Published:** 2018-10-11

**Authors:** Michael Barnett-Cowan, Marc O. Ernst, Heinrich H. Bülthoff

**Affiliations:** 1 Max Planck Institute for Biological Cybernetics, Tübingen, Germany; 2 Department of Kinesiology, University of Waterloo, Waterloo, Canada; 3 Applied Cognitive Psychology, University of Ulm, Ulm, Germany

## Abstract

In environments where orientation is ambiguous, the visual system uses prior knowledge about lighting coming from above to recognize objects, determine which way is up, and reorient the body. Here we investigated the extent with which assumed light from above preferences are affected by body orientation and the orientation of the retina relative to gravity. We tested the ability to extract shape-from-shading with seven human male observers positioned in multiple orientations relative to gravity using a modified KUKA anthropomorphic robot arm. Observers made convex-concave judgments of a central monocularly viewed stimulus with orientations of a shading gradient consistent with being lit from one of 24 simulated illumination directions. By positioning observers in different roll-tilt orientations relative to gravity and when supine, we were able to monitor change in the light-from-above prior (the orientation at which a shaded disk appears maximally convex). The results confirm previous findings that the light-from-above prior changes with body orientation relative to gravity. Interestingly, the results varied also with retinal orientation as well as an additional component that was approximately twice the frequency of retinal orientation. We use a modelling approach to show that the data are well predicted by summing retinal orientation with cross-multiplied utricle and saccule signals of the vestibular system, yielding gravity-dependent biases in the ability to extract shape-from-shading. We conclude that priors such as light coming from above appear to be constantly updated by neural processes that monitor self-orientation to achieve optimal object recognition over moderate deviations from upright posture at the cost of poor recognition when extremely tilted relative to gravity.

## Introduction

To reconstruct the three-dimensional structure of the world, humans use multiple sources of sensory information to estimate environmental properties in addition to relying on prior knowledge to optimally interpret sensory signals^1^. The Bayesian framework has been successful in explaining perceptual phenomena, because it is a statistical framework in which environmental statistics can naturally be incorporated using prior expectations about the world. In this framework, the ambiguities in the sensory information are represented in the likelihood functions, while the prior probability distribution represents pre-existing experience about the statistical regularities derived from the surrounding environment. The posterior probability distribution is proportional to the product of the prior and the likelihood function and is thought to form the basis for the percept. It remains unclear, however, what statistics priors exactly represent or in what reference frames they are coded. One extreme approach would be that environmental statistics are coded in allocentric (world-centred) coordinates such that the priors represent the statistics of the external world irrespective of the sensory receptors with which they are experienced. The other extreme approach may be that environmental statistics are coded in egocentric (receptor-centred) coordinates. In this case the priors would represent the statistics of retinal input (i.e., the statistics of the world convolved with retinal orientation). Alternatively, they may be a mixture of both that might even be flexibly adapted.

Consider for example the light from above prior^2–11^: if this prior represents the light source direction relative to gravity in the world (i.e., the statistics of the external world), the brain would need to discount the orientation of the head, the eyes and the body relative to gravity while learning this prior (world-centred coordinates). On the other hand, the brain may simply encode the statistics of the light source direction as determined on the receptor (here the retina), in which case the statistics are convolved with the eye, body, and head orientations while learning this prior (eye-centred coordinates).

**Figure 1:**
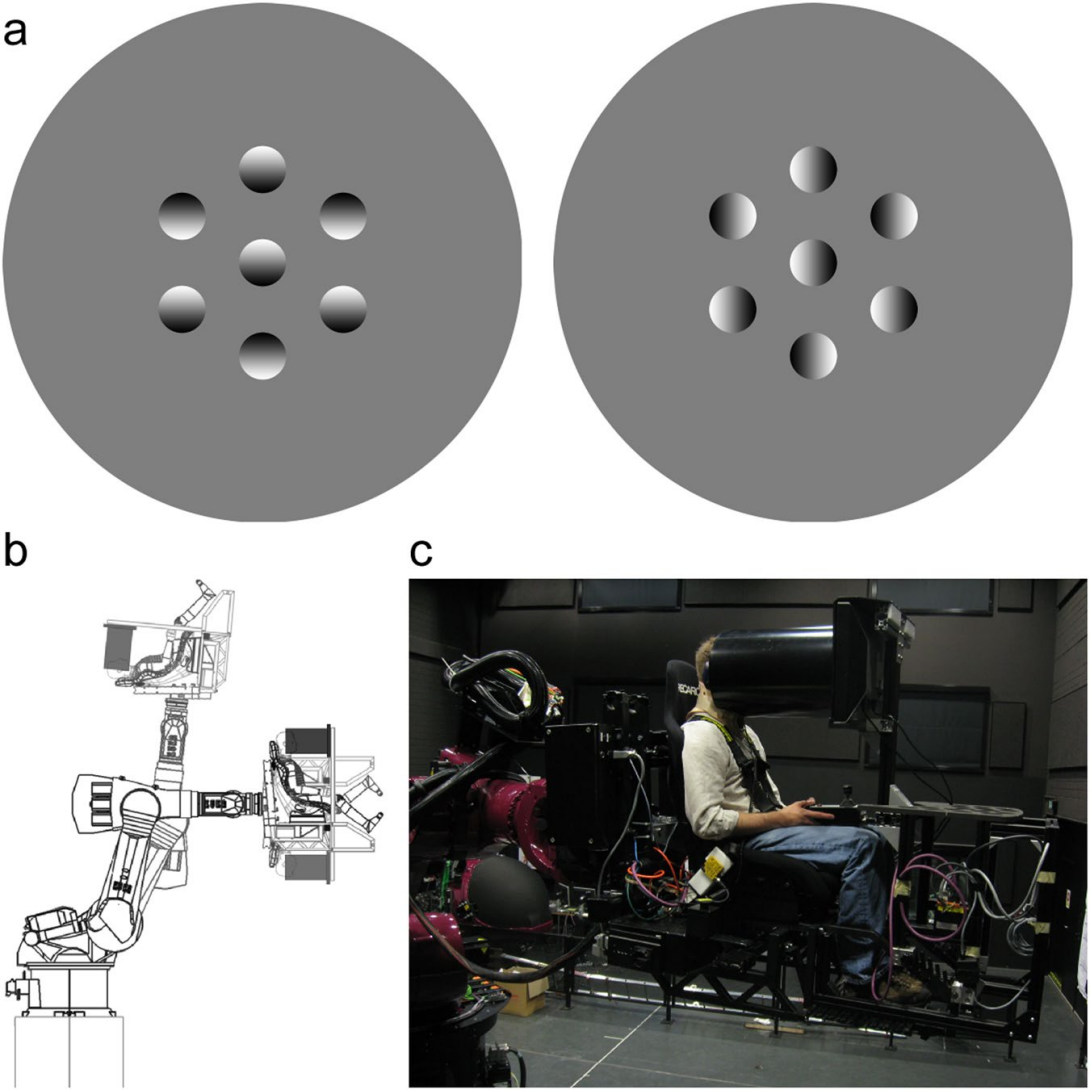
(**a**) Shape-from-shading stimuli. Central patch (3.03° visual angle), consistent with light-from-above (left) flanked by six peripheral patches (11.95° from centre). Another central patch (right) is ambiguous when viewed upright, convex when left-tilted, concave when right-tilted. (**b**) The Max Planck CyberMotion Simulator^9^ positioned observers (upright, supine, and upside-down shown). (**c**) Participant seated with neck brace, harness, and game pad observing stimuli through circular aperture tube in a room that would be dark during testing.

From the literature, we know that in environments where orientation is ambiguous, the visual system uses prior knowledge about lighting coming from above to recognize objects, determine which way is up, and reorient the body^2,3^. The prior assumption that light comes from above can be measured using a shape-from-shading task^4–11^ where assumed lighting direction in the ability to extract shape from shading is predominantly predicted by orientation of the stimulus on the retina (i.e., predominantly eye-centred coordinates). This can be shown using the following demonstration. With one eye closed, the central patch in Fig. 1a (left) appears convex because it and three flankers (which enhance the effect) are brighter (i.e., shaded least) at the top. In Fig. 1a (right), the convex-concavity of the central patch is ambiguous as lighting now comes from the side but when observed by tilting the head to the left, then followed by tilting the head to the right, the central patch appears convex and then concave, consistent with the hypothesis that ‘above’ as regards the ‘light-from-above prior’ does not strictly refer to gravitational above^5–10^. Whether the light-from-above prior is fixed within a particular frame of reference or some combination has been previously explored^5–10^ where it has been shown that the head, the eye, gravity and vision all provide important frames of reference in determining how the light prior (LP) interacts with the stimulus to affect perception. Our objective with this experiment is to better understand the underlying integration process. To do so, we measure the *change* of the LP when observers are positioned in multiple orientations relative to gravity. As the eyes will counter-rotate to be oriented between gravity and the head across a range of body orientations when tilted in the roll plane, this allows us to assess the relative contribution of each of these frames of reference in determining the LP. Further, using this data set we can assess the underlying integration process using a modeling approach with a transfer function description of how known retinal orientation and the response properties of the utricle and saccule signals of the vestibular system may be integrated to resolve shape-from-shading across reference frames.

## Methods

We investigated the relative contributions of head-on-body and retinal orientation relative to gravity in *change* of the light-from-above prior. We tested the ability to extract shape-from-shading with observers positioned in multiple orientations relative to gravity using a modified KUKA anthropomorphic robot arm^12^ (Fig. 1b,c). Seven 21–30 y male observers with normal vision monocularly viewed stimuli (Fig. 1a, left side) from 50 cm through a circular aperture (29.5 cm) in 12 roll postures (0:30:330°) relative to gravity (0°) and when supine (Fig. 1b). Posture order was randomized. Observers made convex-concave judgments of a central stimulus whose shading gradient orientation was consistent with illumination from one of 24 directions (0:15:345°). Each orientation was randomly presented 6 times. Each participant completed 1,728 trials (12 × 24 × 6) that took approximately 2 hours to complete. Trials began with a central green fixation circle (0.53° of visual arc; 1 s), followed by test stimuli (1.5 s), a uniform monochromatic random noise mask stimulus (1 s), and a gray background screen until observer response. Observers gave their informed and written consent prior to their inclusion in the study that the ethics review board of the Max Planck Institute for Biological Cybernetics approved in accordance with the Declaration of Helsinki. Data available from the authors.

**Figure 2:**
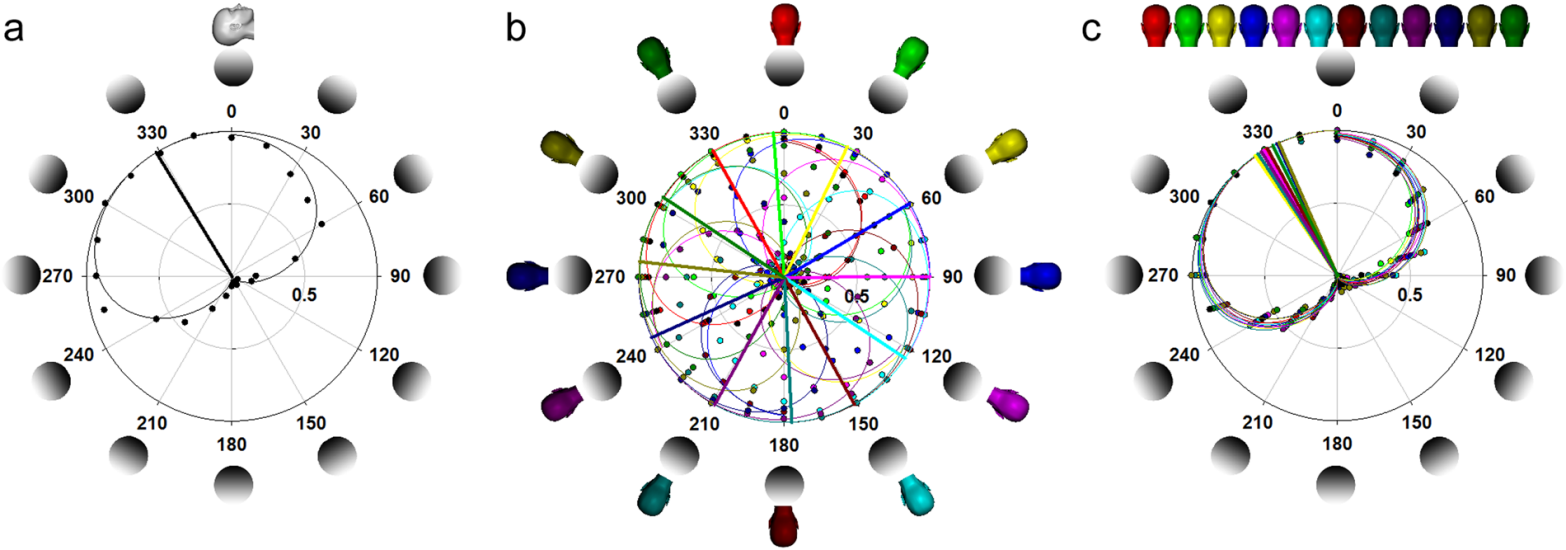
(**a**) Supine LP data (symbolized by head symbol at the top) averaged across all participants. Stimuli lit from above (top of the head) correspond with 0° with values between 0:180° indicating being lit rightward and 180:360° indicating being lit leftward. Here the proportion of stimuli perceived as convex (dots) is fit by two sigmoidal functions (curved lines) each centred at a concave-convex transition whose average gives the peak of the LP (LP_p_ radial line). Note supine data not included in model. (**b**) LP data averaged across participants with the body tilted (symbolized by separate head symbols for each orientation) in gravity coordinates and (**c**) the same data shown relative to head coordinates (right side; mean: 333.5°; range: 328° to 340°**)**.

The peak of the LP (LP_p_) for each participant in each posture was inferred from the data fit (Fig. 2). Here, the percentage of presentations that participants identified the central stimulus as ‘convex’ was plotted as a function of the lighting direction that the central stimulus was lit from. Two sigmoidal functions (Equation 1) were fitted to the participants’ response rate using Sigmaplot v12, which uses a residual sum of squares curve fitting approach to determine each of the convex-to-concave and concave-to-convex transitions for each body orientation. Here one sigmoid function was fit to the descending data points (convex to concave) and the other was fit to the ascending data points (concave to convex), with the two sets of data points divided by hand.

1 $$y=\frac{100}{1+e\frac{x-x0}{{\rm{\sigma }}}} \% $$

where: x_0_ corresponds to the 50% point and σ is the standard deviation. The average of the orientations at which these two transitions occurred was taken as the LP_p_.

## Results

**Figure 3:**
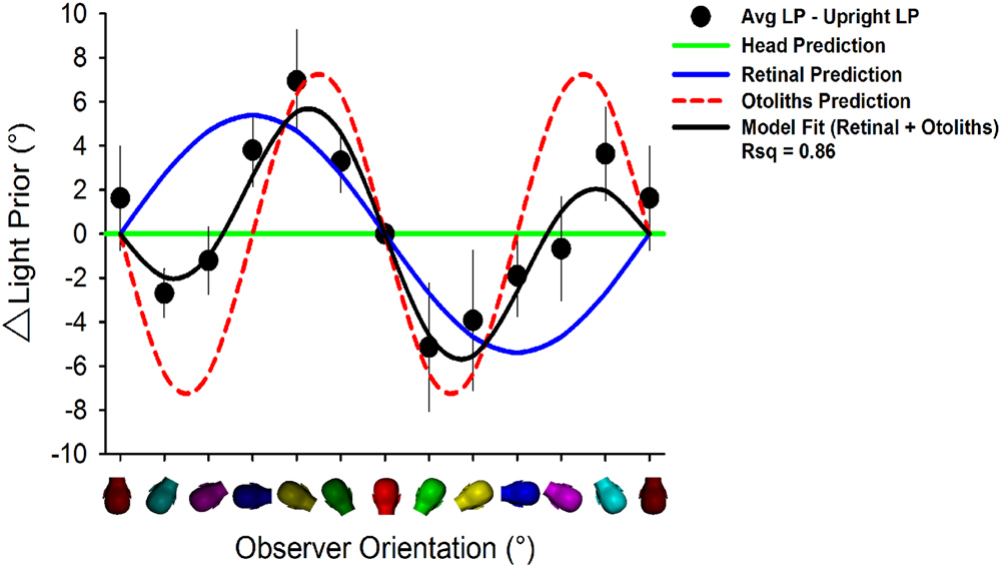
Change in the LP_p_ averaged across participants (black symbols) relative to upright posture (0° on abscissa; red head symbol) for roll tilt of the observer. Prediction for change in the LP_p_ from head orientation (i.e., no change; green horizontal prediction line), retinal orientation (blue prediction line; from^13^), otoliths (red prediction line; see Fig. 4), or the retinal + otolith model (black prediction line; see Fig. 4). Error bars, +/−1 s.e.m.

When supine, where the influence of gravity and torsional eye movement are nulled, the LP_p_ averaged across participants was up and significantly to the left of the head by −31.9° (s.e.: 8.7; one-sample t-test: t(6) = 3.5, p = 0.012, β = 0.839; Normality (Shaprio-Wilk): passed, p = 0.832; see Fig. 2a). Figure 2b shows the LP_p_ averaged across participants for each body orientation in gravitational coordinates, whereas Fig. 2c shows these same data in body coordinates. As the purpose of our experiment was to assess gravity-dependent *change* in the LP_p_, we measured the LP_p_ in each body orientation and subtracted this from the LP_p_ that was measured with the observer upright relative to gravity so to express the LP_p_ in head-centered coordinates (Fig. 3). This was done not only to look at *change* in the LP_p_ relative to a baseline upright posture, but also to account for the large individual differences in the LP_p_ leftward bias. Within this head-centered reference frame, the utricle would give zero response (sin(0) = 0) and the saccule would be maximal (cos(0) = 1) when the observer is upright. This is the 0 point on the abscissa where all observers are truly upright and *change* of the LP_p_ relative to the LP_p_ when upright collapses to zero (i.e., no change). Thus, if the LP_p_ has a tilt of 10° to the left relative to gravitational ‘above’ when upright then any *change* of the LP will be made relative to this offset bias (i.e., 0 *change* when upright).

**Figure 4:**
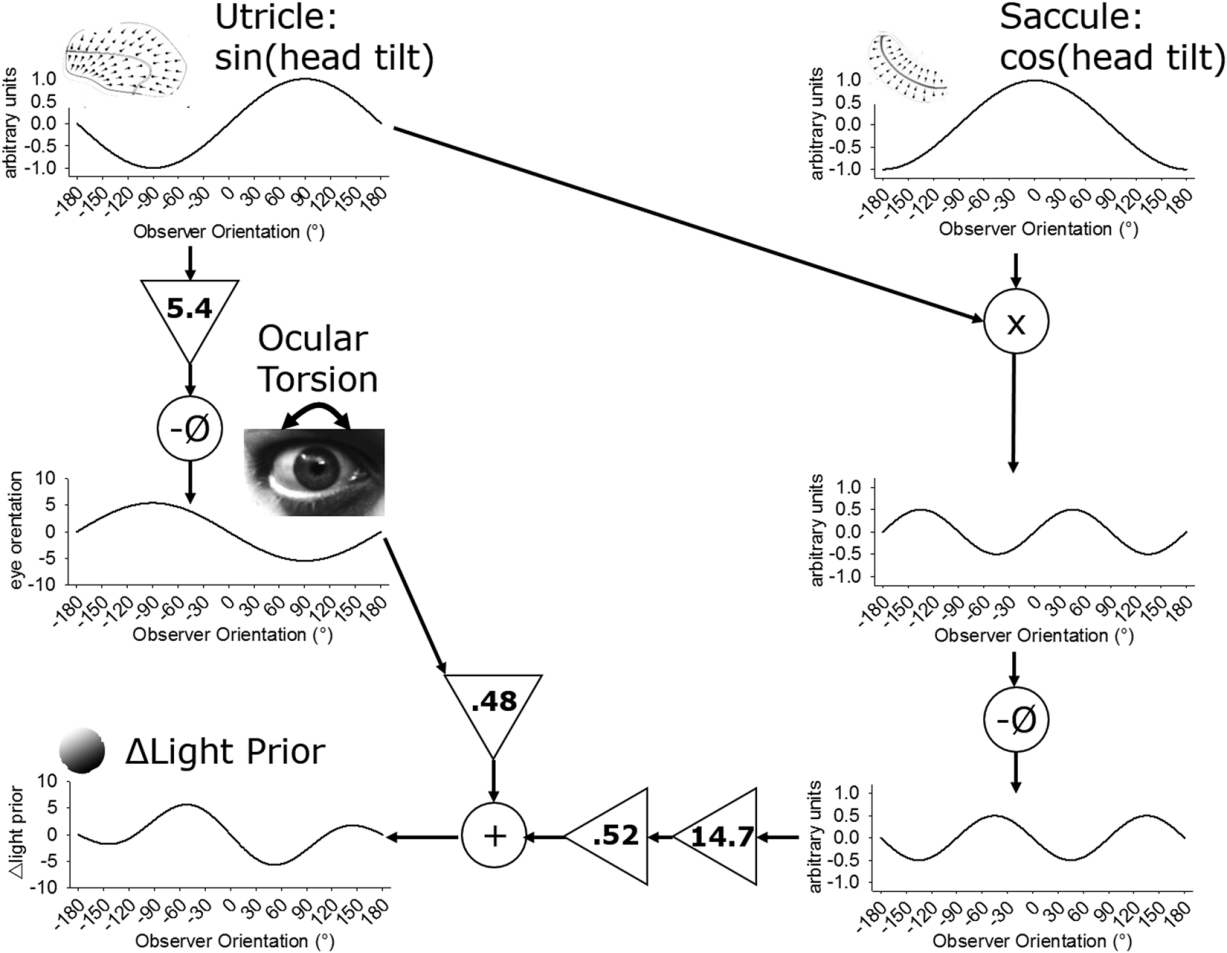
Theoretical model (Equation 3) showing how the inverse (−Ø) product (x symbol) of utricle (sin(head tilt)) and saccule (cos(head tilt)) signals with a gain of 14.7 are summed with retinal orientation (5.4° * −sin(ø)) with near equal weights (0.52 and 0.48, respectively) to predict change in the change in LP_p_ for roll tilt of the observer. Retinal orientation prediction from^13^. The amplitude of multiplied otolith signals and weighting relative to retinal orientation are free parameters.

Significant change of the LP with posture rules out that the LP_p_ is fixed to the head (RMANOVA: F_(12,72)_ = 3.2, p = 0.001, β = 0.925; Normality (Shapiro-Wilk): passed, p = 0.825; Equal variance: passed, p = 0.091; Fig. 2b radial lines). When the head tilts relative to gravity, the eyes partially counter-rotate in order to maintain perceptual stability. Thus, if the LP were fixed to the retina, data in Figure 3 would vary sinusoidally by Equation 2^13^ (blue line, R^2^ = 0.28, p = 0.06). We find however that change of the LP_p_ is best fitted by the near equally weighted sum of the product of utricle (i.e., sin(head tilt)) and saccule (i.e., cos(head tilt)) responses to tilt of the head relative to gravity^14,15^ (red line) added to retinal orientation^13^ (Fig. 4; black line in Fig. 3; R^2^ = 0.94, p < 0.001; Equation 3).

2 $$y={5.4}^{^\circ }\ast -\,\sin (\varnothing )$$

3 $$y={w}_{o}\,(k\ast -(\sin (\varnothing )\ast \cos (\varnothing )))+{w}_{e}\,({5.4}^{^\circ }\ast -\,\sin (\varnothing ))$$

where: *y* is change of the LPp, ø is head tilt relative to gravity, *k* is a gain, and *w*_*o*_ and *w*_*e*_ are weights attributed to the otolith and retinal orientation relative to gravity components, respectively.

A Bayesian Information Criterion (MATLAB® 2013; aicbic function) was then calculated comparing our model with two free parameters (constant gain, relative weights; Equation 3, Fig. 3 black line, Fig. 4) with the simple parameter-free eye in head model (Equation 2, Fig. 3 blue line) which yielded a ΔBIC score of 132.154 in favour of Equation 3 over Equation 2, which is considered to be a “very strong” difference^16^. This suggests that participants’ estimates of shape from shading are better predicted with a retinal orientation model plus the inclusion of an additional component, which we attribute to cross multiplied otolith signals as depicted in Fig. 4.

## Discussion

That the LP represents a bias of light coming from above and to the left of the head both when supine and when positioned in various roll-tilt body orientations relative to gravity is consistent with previous psychophysical^6,8,9,11,17^ and neuroimaging reports^18^. Although the supine data suggest that the leftward bias is largely within the head-on-body frame of reference, our results also confirm previous work that this prior is not invariant when the body tilts relative to gravity (i.e., it does not reside solely in a unique frame of reference)^5–10^. Rather the likelihood function that represents sensory information about light coming from above and to the left *changes* with incoming orthogonal vestibular responses to tilt of the head relative to gravity. One distinguishing feature of our results is that while we confirm the results of others that gravity can change the LP^5–9^, our methods and modelling approach are able to distinguish gravity’s differential influence on ocular torsion of the eye versus change in central processing of visual shape from shading information.

It has been previously found that LP data are consistent with a combination of both retinal and gravitational frameworks, where the LP is largely predicted from retinal orientation plus an additional gravitational component^9^. It is important to note that the retinal model for predicting change of the LP with tilt of the body relative to gravity fit to data in the present experiment is quite robust for small changes in orientation (i.e., 0–60° such as in^9^), where retina-based and head-based predictions are more similar than at extreme tilt angles. What distinguishes our results is the large range of observer tilt angles used to test shape from shading perception with the body extremely tilted to better understand deviations from the retinal prediction. Here, when nearly upside-down (e.g, 150°) predictions for head and retina-based frames of reference fail to predict change in the LP from an initial upright posture. Consequently, because the light-from-above prior is updated when extremely tilted relative to gravity and not aligned with gravity anymore, errors in object recognition may increase as the light is still likely to come from the gravity upright direction.

One interpretation of our results put forward in our model is that otolith signals are multiplied and added to visual information in a retinotopic reference frame. There is, however, an alternative explanation for our results. Our model works on the assumption that the direction of the light-from-above prior can be represented as a weighted sum of sin(theta) * cos(theta) and sin(theta) terms. As the first term is equivalent to sin(2 * theta), an alternative equation of the data can be represented by the first two sinusoidal harmonics. As both equations are possible, future work will be required to determine the underlying neurophysiological determinants. Given that the data has to go through the point (0,0) and is antisymmetric (i.e., has opposite signs on either side of theta = 0), the alternative explanation of the data being represented by the first two sinusoidal harmonics is entirely plausible and accordingly tell us little about the underlying physiology on its own. We are in favour of interpreting the results of our experiment as likely being the sum of cross-multiplied utricle and saccule signals added to the retinal component based largely from supportive literature for such a mechanism. Multiplication has been suggested to play a role in many aspects of neural computation^14,19^. Response properties consistent with multiplication have been observed in auditory neurons of the barn owl where multiplication of separate postsynaptic potentials tuned to interaural time and level differences form a coherent representation of auditory space^20^. Neural recordings that support cross-multiplication of vestibular signals at the level of the vestibular nucleus have also been reported^21^. Neurons in monkey posterior parietal cortex exhibit gain field properties that can be explained by a multiplication of retinal and eye or head position signals^22^ and functional magnetic resonance imaging experiments in humans have revealed that neurons in this region also decode shape-from-shading^18^ and respond to vestibular stimulation^23^. Finally, not only is there a physiological basis for suggesting that cross multiplication of otolith signals is biologically plausible, there is reasonable cause for the central nervous system to perform these transformations. To wit, as perceptual measures of verticality also suggest these multiplicative response properties^14,24^, we suggest that cross-multiplication of otolith signals may be used to update the representation of 3D space in parietal cortex. Future neurophysiological work is thus required to assess whether cross multiplication of otolith signals are added to visual information in a retinotopic reference frame or alternatively whether the added component is derived from the first two sinusoidal harmonics of otolith signals.

In conclusion, priors such as light coming from above appear to be constantly updated by neural processes that monitor self-orientation to achieve optimal object recognition over moderate deviations from upright posture at the cost of poor recognition when extremely tilted relative to gravity. This interpretation is consistent with numerous other studies, which indicate that the brain constructs an internal representation of the body with a prior assumption that the head is upright^14,17,25–30^. Previously it was suggested that the gravitational frame of reference might become insignificant when an observer is upside-down^7^. Within this framework, robust cue-combination can be found for small conflicts between stimuli, but when larger conflicts appear (such as when observers are upside–down) then one frame of reference is expected to dominate. Our results support this approach but also provide additional insight into the possible source for the gravitational cue being cross-multiplied utricle and saccule signals of the vestibular system, yielding gravity-dependent biases in the ability to extract shape-from-shading.
